# Combined selective portal vein embolization and excluded bile duct sclerotherapy (SPEEDS) in the treatment of excluded bile ducts

**DOI:** 10.1007/s00423-026-04004-7

**Published:** 2026-04-01

**Authors:** Niclas Dohrn, Katrine Folmann Finne, Susanne Frevert, Ruben J. Jensen, Christoph Tschuor, Jan Henrik Storkholm, Peter Nørgaard Larsen

**Affiliations:** 1 Department of Digestive Diseases, Transplantation and General Surgery, Copenhagen University Hospital - Rigshospitalet, Inge Lehmanns vej 7, Copenhagen, DK-2100 Denmark; 2 Department of Radiology, Copenhagen University Hospital - Rigshospitalet, Inge Lehmanns vej 7, Copenhagen, DK-2100 Denmark; 3 University of Copenhagen, Blegdamsvej 3, Copenhagen, DK-2200 Denmark

**Keywords:** Excluded bile ducts, High-output biliary fistula, Selective portal vein embolization, Sclerotherapi

## Abstract

**Background:**

Bile leakage from an excluded bile duct is a rare complication after hepatobiliary surgery. It can cause high-output biliary fistulas with high morbidity or even death. Traditionally, management of this condition has been surgical with liver resection or hepaticojejunostomy. To avoid these high-risk salvage surgeries, minimally invasive procedures have been proposed, but with conflicting results. We hypothesized that a staged combination of both percutaneous transhepatic Selective Portal vein Embolization and Excluded bile Duct Sclerotherapy (SPEEDS) is a feasible, safe, and effective therapy for these patients.

**Methods:**

This is a retrospective single-center study from a high-volume hepato-biliary center. We reviewed all consecutive patients with bile leakage from excluded bile ducts from April 2018 until September 2024.

**Results:**

We identified nine patients with high-output bile leakage from excluded bile ducts during the study period. All nine patients were managed with the novel SPEEDS technique. The treatment was successful, with complete symptom resolution in all patients and no serious adverse events.

**Discussion:**

SPEEDS is a feasible, safe, and effective therapy for patients with bile leakage from excluded bile ducts. Further research and consolidation of the technique are needed.

## Introduction

Postoperative bile leakage following liver resections occurs with an incidence of 4–9% [1–7]. Postoperative bile leakage from bile ducts severed from the remaining biliary tree but still draining parts of the functional liver remnant is a rare type of bile leakage occurring after only 0.1–0.5% of hepatectomies [1, 2, 5, 6]. It can cause persistent high-output biliary fistulas (HOBF) [4], which may lead to fluid and electrolyte imbalances, malnutrition, extensive and prolonged morbidity, including intraperitoneal septic complications, post-hepatectomy liver failure, prolonged hospitalization, and even death [2, 4].

HOBF from bile ducts excluded from the rest of the biliary tree has little potential for spontaneous closure [1, 2, 4–6, 8, 9], and the traditional treatment for biliary leakage with drainage and biliary decompression with endoscopic sphincterotomy has no role in this condition because of the lack of communication to the rest of the biliary tree [1, 7]. Therefore, surgical treatment of this condition includes salvage surgery with liver resection or diversion with hepaticojejunostomy. However, these procedures are performed in hostile abdomens with dense adhesions from biliary peritonitis and are at a high risk of complications [1, 2, 4–8]. To avoid these high-risk procedures, minimally invasive procedures such as either percutaneous transhepatic biliary drainage (PTBD) with subsequent bile duct sclerotherapy and/or percutaneous transhepatic Selective Portal Vein Embolization (SPVE) have been proposed but only limited described [2, 5, 9–15].

We hypothesized that a staged combination of these therapies, including percutaneous transhepatic Selective Portal vein Embolization and Excluded bile Duct Sclerotherapy (SPEEDS), is a feasible, safe, and effective therapy for patients with excluded bile ducts. This paper aimed to describe the technical aspects of our treatment strategy with SPEEDS and evaluate the initial results.

## Patients and methods

This observational study describes the technical aspects of the treatment and the results of nine consecutive patients with excluded bile ducts treated with SPEEDS at a tertiary, high-volume hepatobiliary center during an exploratory phase.

### Definition of bile leakage

We defined bile leakage after hepatectomy according to the International Study Group of Liver Surgery: drainage of fluid with a bilirubin level three times the serum level on postoperative day 3 or later, or the need for interventions due to biliary collections or peritonitis [16].

Bile leakages are classified into four categories by Nagano et al. [1]:

#### Type A

Minor leakage from the cut surface, with radiology showing no bile duct involvement and only a small amount of bile leakage.

#### Type B

Major leakage due to insufficient closure of a bile duct stump connected to the remaining bile duct three.

#### Type C

Major leakage due to lateral injury of the bile duct.

#### Type D

Bile leakage due to complete division of a bile duct with a peripherally excluded bile duct draining functional liver parenchyma.

### Diagnosis of high-output biliary fistula (HOBF) type D

The diagnosis was based on the findings of:

#### 1. HOBF

Drainage of > 100 ml of fluid per day from the abdomen with > 3 times the serum level of bilirubin persisting at least one week postoperatively.

#### 2. Type D bile leakage

Evidence of an excluded bile duct and biliary leakage/fistula from a contrast-enhanced MRI, later confirmed with percutaneous transhepatic cholangiography (PTC).

### Patients

From 2019 to 2024, all consecutive patients diagnosed with HOBF Nagano type D leakage were referred to treatment at the interventional radiology department and treated with SPEEDS. Patients were identified from a prospectively recorded database, but medical records were retrospectively reviewed. Complications were recorded and classified according to the Clavien-Dindo classification (CDC) [17]. The nomenclature of segments and types of liver resection follows the Brisbane 2000 terminology [18].

### Management technique

#### Combined staged percutaneous transhepatic selective portal vein embolization and excluded bile duct sclerotherapy (SPEEDS)

SPEEDS is a staged combination therapy with percutaneous transhepatic selective portal vein embolization and percutaneous transhepatic cholangiography drainage (PTCD) of the excluded bile ducts, including subsequent sclerotherapy. The method has yet to be defined and described, and in this paper, we describe our approach to this treatment modality. During the duration of this study, we altered the technique based on our experiences.

In all patients, biliary diversion was initially performed by placing a PTCD into the excluded bile duct, essentially also confirming the diagnosis of Type D bile leakage. Subsequently, a percutaneous transhepatic selective portal vein embolization (SPVE) with NSPVA particles (350–500 μm) and sometimes vascular plugs was performed in the corresponding segmental portal vein.

**Fig. 1:**
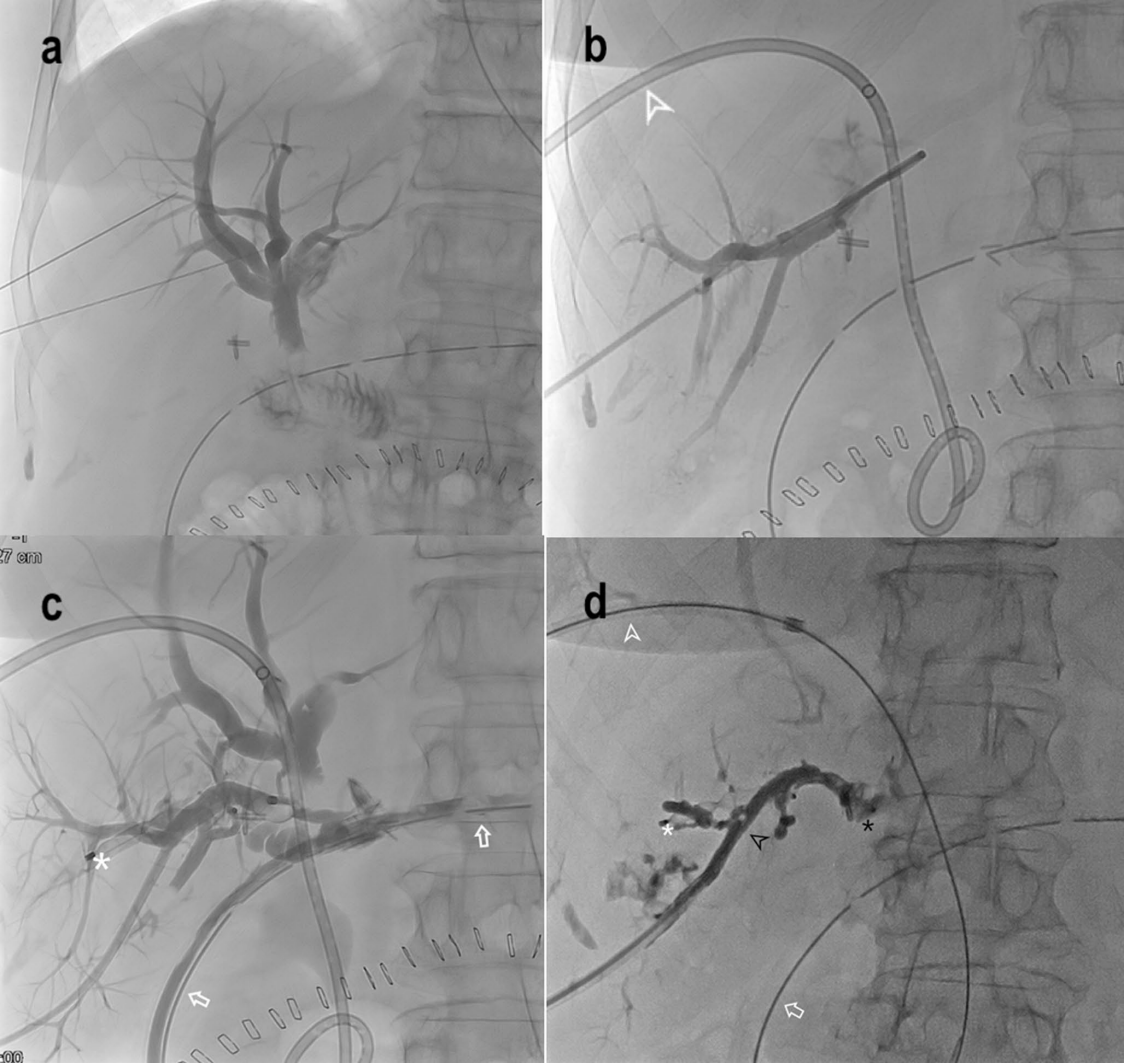
SPEEDS procedure in patient #5 with pancreatic cancer who underwent surgery with pancreaticoduodenectomy. Diagnosis of Nagano type D bile leakage of a segment 6 bile duct was done with Primovist-enhanced MRCP (not shown in the figure). **a** POD 3: PTC before placement of internal/external drain. There was no leakage from the hepaticojejunostomy. **b** POD 18. The portal vein branch of the excluded liver segment before embolization. 8.5 F internal/external PTC drain marked with white arrowhead. **c** POD 18: PTC before placement of an 8.5 F external drain in the excluded bile duct. Leakage from the excluded bile duct to the peritoneal cavity and into the surgical drain (white arrows). Plug from portal vein embolization is marked with a white asterisk. **d** POD 31. Glue cast (black arrowhead) during sclerotherapy of the excluded bile ducts on POD 31. A plug (AVP II) (black asterix) was placed before sclerotherapy injection. SPEEDS: Selective Portal vein Embolization and Excluded bile Duct Sclerotherapy; POD: Postoperative day; MRCP: Magnetic resonance cholangiopancreatography; PTC: Percutaneous transhepatic cholangiography

After SPVE, sclerotherapy of the bile ducts was conducted by instillation of 3–5 ml of alcohol and finally casted with glue using the external PTCD. Timing or sclerotherapy was variable. If the fistula communicated with a cavity at the resection margin, the alcohol was isolated in the bile duct using an Amplatzer Plug^®^ glued with a Histoacryl^®^/Lipiodol^®^ mixture at the exit to the cavity or by a temporarily inflated Fogarty-catheter in the cavity pulled towards the bile duct. The alcohol was kept in place for 5 min and afterwards aspirated. Sclerotherapy was repeated every 3rd – 7th day with variations between patients. Treatment was continued until the HOBF output was < 50 ml/day, or the drain fluid bilirubin concentration was < 50 µmol/L. The PTBD was then removed without preceding clamping (Fig. 1).

### Statistical analysis

Descriptive variables were calculated for the overall cohort: categorical variables were expressed as numbers (percentages), while numerical variables were expressed as median [range].

## Results

Nine patients with HOBF Nagano type D were treated with SPEEDS from 2019 to 2024. The median age was 67 [37–78]; five were male, and four were female. None of the patients had underlying liver disease (e.g., cirrhosis).

Table 1 presents the nine patients in the order in which they presented to our center. In 67% (*n* = 6) of the patients, index surgery was performed due to malignant disease (cholangiocarcinoma (*n* = 4), pancreatic cancer (*n* = 1), and colorectal metastasis (*n* = 1)). In the remaining 33% (*n* = 3), surgery was done due to benign disease with cholecystolithiasis (*n* = 2) and a benign duodenal tumor (*n* = 1). In 89% (*n* = 8) of the patients, the leakage was from the right liver (segments 5–8) and, in one patient (11%), from lobus caudatus (segment 1).

**Table 1:** Selective portal vein embolization and excluded bile duct sclerotherapy (SPEEDS) patients in sequential order

Nr.	Indication for primary surgery	Primary surgery	Issue	Leaking excluded liver segment	Days until SPEEDS from index surgery	Daily fistula output before SPVE (mL)	Number of sclerotherapies	Days between sclerotherapies	Daily fistula output before last sclerotherapy (ml)	Duration of sclerotherapy (days)	Complete duration of SPEEDS (days)	Treatment successful	Follow-up (months)
#1	Distalt cholangiocarcinoma	Open Whipples procedure	Blumgart C2 configuration. Division bile duct from S6	Segment 6	50	800	3	2 to 3 days	50	5	209	Yes	56
#2	Cholecystolithiasis	Laparoscopic cholecystectomy	Division of bile duct from S5	Segment 5	20	300	7	3 to 7 days	20	28	106	Yes	57
#3	Liver metastasis colorectal cancer	Open segment 1 resection	Division of bile duct from S1	Segment 1	70	60	5	7 days	5	28	39	Yes	57
#4	Hilar cholangiocarcinoma	Open Klatskins procedure with left hepatectomy	Division of bile duct from S7	Segment 7	23	300	1	-	25	1	26	Yes	45
#5	Pancreatic cancer	Open Whipples procedure	Blumgart C2 configuration. Division of bile duct from S6	Segment 6	18	300	1	-	50	1	13	Yes	34
#6	Intrahepatic cholangiocarcinoma	Open Klatskins procedure with left hepatectomy	Division of bile ducts to posterior sector	Segment 6 & 7	18	950	1	-	250	1	23	Yes	17
#7	Tumor of the duodenum	Open Whipples and segment 5 resection	Division of bile duct from S5	Segment 5	18	400	5	4 to 365 days	25	377	385	Yes	3
#8	Intrahepatic cholangiocarcinoma	Open extended left hepatectomy	Remaining segment 8 with excluded bile duct.	Segment 8	94	300	2	9 days	5	9	9	Yes	6
#9	Acute calculous cholecystitis	Laparoscopic cholecystectomy	Blumgart C1 configurration. Division of anatomic variant of bile ducts S5 + S6	Segment 5 & 6	25	350	2	2 days	150	2	4	Yes	1

*SPEEDS*: Selective Portal vein Embolization and Excluded bile Duct Sclerotherapy; *SPVE*: Selective portal vein embolization

SPEEDS treatment was successful in all patients (9/9). The median follow-up after termination of sclerotherapy was 26 months [1–56 months]. None of the patients had a recurrence of HOBF or required salvage surgery. No severe adverse events were experienced. Three patients died during the follow-up period, but all deaths were attributed to other causes.

One patient (#6) developed a CDC grade IIIa complication with a liver abscess in the atrophied liver segment and an infection around a displaced vascular plug. The complication was handled successfully with percutaneous drainage and antibiotics in the outpatient clinic.

Table 2 presents our summarized results with SPEEDS. The median duration of SPEEDS was 26 days, of which 13 days constituted the time between SPVE and 1st sclerotherapy. The combined approach with SPEEDS treatment reduced the fistula output from a median of 300 to 25 mL/day and the bilirubin concentration from a median of 323 to 36 µmol/L whereas SPVE alone reduced the median output from 300 to 150 mL/day.

**Table 2:** Metrics of SPEEDS

	Median	Range
Duration from index surgery until initiation of SPEEDS (days)	23	[18–94]
Duration between SPVE and sclerotherapy (days)	13	[0–204]
Total number of sclerotherapies (*n*)	2	[1–7]
Duration of sclerotherapy (days)	5	[1–377]
Total duration of SPEEDS (days)	26	[4–385]
Daily output from HOBF before PVE (before SPEEDS) (mL)	300	[60–950]
Daily output from HOBF before 1st sclerotherapy (mL)	150	[5–300]
Daily output from HOBF before last sclerotherapy (mL)	25	[5–250]
Bilirubin concentration from HOBF before SPEEDS (µmol/L)	323	[115–1300]
Bilirubin concentration from HOBF before last sclerotherapy (µmol/L)	36	[3–225]

*SPEEDS*: Selective Portal vein Embolization and Excluded bile Duct Sclerotherapy;* SPVE*: Selective portal vein embolization;* HOBF*: High-output biliary fistulas

## Discussion

This paper describes the initial results and technical aspects of a novel minimally invasive treatment strategy for patients with excluded biliary bile ducts with a combined and staged treatment with Selective Portal vein Embolization and Excluded bile Duct Sclerotherapy (SPEEDS). We found that the technique is safe, feasible, and without any serious adverse events. We also found that the efficacy of the treatment was high, with 100% of the patients treated successfully.

The modalities of bile duct sclerotherapy and selective portal vein embolization for the management of type D leakages have previously been described in case reports with limited numbers of patients, inconsistent results, and often as monotherapy [2, 5, 7, 9–13, 15, 19]. Using the modalities combined and staged (SPEEDS) for these patients is novel. It has only been described as a combined concept once in a case report (*n* = 1) by Kubo et al. [10]. They described a patient with a HOBF with an output of 200 mL/day, and because of the high output, monotherapy with SPVE or bile duct sclerotherapy was considered inadequate, and a staged combination was planned. After SPVE, output was reduced to 50 mL/day. Two weeks later, alcohol sclerotherapy in the excluded bile duct was initiated and repeated one week later, and the output was reduced to < 10 mL/day. The treatment was successful, without recurrence or adverse events. The approach described by Kubo et al. inspired us to initiate the management with SPEEDS [10].

Sadakari et al. [11] described a patient with type D bile leakage initially treated with drainage and four repetitions of alcohol sclerotherapy two months after index surgery reducing output from 100 to 50 ml/day. However, the fistula was not under control, and SPVE was performed additionally two months after the index surgery. SPVE was successful with liver atrophy and termination of the fistula. This was an unplanned combination of sclerotherapy and SPVE. In the SPEEDS protocol, SPVE precedes sclerotherapy to reduce bile production of the excluded part of the liver before closing the bile ducts with sclerotherapy, but this case indicates that the order might not be important. In our latest patient (#9), we successfully performed SPVE and sclerotherapy simultaneously, which resulted in an effective treatment with a total duration of SPEEDS of only four days and two treatments. This is very cost-effective, but further research is needed to validate the benefits of simultaneous SPVE and sclerotherapy.

Shimizu et al. [13] reported a patient with a type D bile leakage with an output of 150 ml/day successfully managed with monotherapy of alcohol sclerotherapy alone. The sclerotherapy regimen was intense, with five treatments each week and a total of 23 treatments. Matsumoto et al. [12] described a similar patient with successful alcohol sclero-monotherapy, with one treatment each week and a total of 11 treatments. Kyokane et al. [15] reported a patient with successful alcohol sclerotherapy with two treatments each week and a total of 6 treatments. Even though all were eventually successful, monotherapy seems ineffective and resource-demanding. We observed a significant effect of SPVE, with a reduction in daily output from a median of 300 to 150 mL/day before the 1st sclerotherapy. Only two treatments with sclerotherapies were needed thereafter, demonstrating the potential benefit of a multimodal approach. In the initial case report by Kubo et al. [10], a similar reduction in output after SPVE from 200 to 50 mL/day was observed.

Tanaka et al. [5] described two patients with excluded bile ducts who were successfully treated by sealing the ducts with a mixture of fibrin glue and iodized oil. However, it is notable in these patients that output had already spontaneously reduced to less than 50 ml/day, 50 and 78 days after index surgery. We only used sealant as an adjunct to sclerotherapy to prevent collateral damage with alcohol going into the free peritoneum; however, the results suggest that sealant could be incorporated into the primary management therapy in patients with aseptic and low-output biliary fistulas (< 50 ml/day).

Honore et al. [4] described a patient with excluded bile ducts in whom HOBF output was reduced from 200 to 100 ml/day with SPVE. With additional arterial embolization, the output could be reduced to 80 ml/day. No necrotic complications were described following this double embolization technique. It is worth speculating if arterial embolization has a role as an adjunct to the SPEEDS treatment in refractory cases. However, caution is warranted in patients with bilioenteric anastomosis, where the risk of septic complications may be increased.

The present description of management with SPEEDS is based on non-empirical assumptions on the most effective regimen (timing, frequency, dosage, sclerotherapy, criteria for drain removal, etc.), and we made small adjustments during the duration of the study, e.g., the interval between the sclerotherapies (3–7 days), and the interval between the different modalities. The SPEEDS technique may be modified to an even more cost-effective regimen.

The total duration of SPEEDS treatment was a median of 26 days, of which 13 days were between SPVE and the continuation with sclerotherapy. We managed the patients in the outpatient clinic, and therefore, the treatment has the potential to significantly reduce the burden on the healthcare system compared to the alternative with complex salvage surgery followed by a long admission [4]. Kimura et al. [14] described a non-surgical approach for peripheral bile leakage, treating 10 patients with external drainage alone. They found that the treatment was successful in 7/10 patients (ethanol sclerotherapy had to be added in one case, and two died during the treatment), but the treatment duration was very long (mean duration of drainage of 157 ± 132 days). Prolonged duration not only carries a substantial burden on the healthcare system but also carries a substantial burden on patients with long-term morbidity. With a duration of SPEEDS treatment of 26 days, this indicates that adding SPVE and repetitive sclerotherapies to external PTCD increases effectiveness compared to drainage alone.

One patient in our series (#7) had a longer duration of SPEEDS treatment of 385 days. The patient initially had SPVE followed by two repetitions of sclerotherapy. Unintentionally, the external PTCD in the excluded bile duct was removed when drainage output was still high (around 150 ml), and the SPEEDS treatment was therefore discontinued before the target output of < 50 ml/day was achieved. We decided on conservative management from this point, but the patient was later referred with biloma and cholangitis. Subsequently, we decided to continue the SPEEDS with a one-year delay and three additional sclerotherapies. This case confirmed the strategy of obtaining a target for daily output before discontinuing SPEEDS.

We found a median delay of 23 days from index surgery until the diagnosis of a type D bile leakage was made and treatment with SPEEDS was initiated. This diagnostic delay is in accordance with the literature, where a delay of 1–9 months is described before diagnosis [9, 11–14]. The diagnosis is typically delayed because type D bile leakage is rare and therefore not suspected initially, and because most bile leakages are self-limiting and treated with simple drainage alone [1, 2, 11, 14]. During a diagnostic delay, the patients often have septic complications with biliary peritonitis with a need for biliary diversion with percutaneous drainage from the peritoneal cavity [2]. This delay can complicate salvage surgery and emphasizes the need for (1) early suspicion of the diagnosis with early MRI and (2) an effective minimally invasive approach for managing these patients.

Previous papers only describe patients with excluded bile ducts following liver resections [1, 2, 4, 5, 7–14]. However, in our case series, excluded bile ducts also occurred after complicated laparoscopic cholecystectomy or pancreaticoduodenectomy, where the damage occurred during skeletonization of the hepatoduodenal ligament at the hepatic hilum in combination with anatomical variations. This emphasizes the need for careful dissection of the hepatoduodenal ligament and focus on early recognition of anatomical variations [8], but also that the complication applies to procedures other than hepatectomies.

Previous studies [1, 2, 4–6, 8] have described the management of patients with type D bile leakage using salvage surgery. Even though the papers state that the management was successful, the surgeries were also complex, with extensive adhesiolysis, operative times of more than 5 h, average blood loss of more than 1,5 L, and a length of stay after reoperation of 10–30 days. We argue that there is a need for a minimally invasive non-surgical solution for these patients because salvage surgery comes with a high risk [1, 2, 4–6].

The limitations of this study are the retrospective and non-comparative design. This paper suggests that the technique is feasible, safe, and effective, but ideally, a randomized controlled trial should be performed to draw firm conclusions on superiority over other treatment modalities. However, type D bile leakage is a rare complication (0.1–0.5%) [1, 2, 4, 5], and the feasibility of an RCT on this condition is doubtful. Clinical practice will likely have changed before the results of such a trial ever become available. There is no comparative prospective or retrospective evidence supporting either a minimally invasive or a surgical approach. In addition, there is currently no comparative evidence supporting the combined approach versus monotherapy with selective portal vein embolization or bile duct sclerotherapy alone. The final limitation is that a rigorous SPEEDS approach was not protocolized, and we made modifications along the sequence of the case series based on experiences (e.g., the intervals between sclerotherapies and the interval between SPVE and sclerotherapies) (Table 2).

The strength of this study is that all consecutive patients with type D bile leaks were treated with the intervention, thereby limiting selection bias. The study was performed as a single-center study and by a small number of interventional radiologists, which increased the standardization of the approach. Finally, because of the rarity of the condition, only case reports or small case series on interventional treatment options are available in the literature; this paper contributes the largest case series of patients with type D bile leakage.

## Conclusion

High-output biliary fistula caused by excluded bile ducts is a rare and complex complication. Management with combined staged Selective Portal Embolization and Excluded bile Duct Sclerotherapy (SPEEDS) is safe and feasible, thereby offering an effective minimally invasive approach for these patients. However, further research and consolidation of the SPEEDS technique are warranted.

## Data Availability

No datasets were generated or analysed during the current study.

## Declarations

### Commentary

The paper is not based on a previous communication to a society or meeting.
